# Evaluation of BCL6 and SIRT1 as Non-Invasive Diagnostic Markers of Endometriosis

**DOI:** 10.3390/cimb43030096

**Published:** 2021-09-28

**Authors:** Alison M. Sansone, Brooke V. Hisrich, R. Brandt Young, William F. Abel, Zachary Bowens, Bailey B. Blair, Avery T. Funkhouser, David P. Schammel, Lisa J. Green, Bruce A. Lessey, Anna V. Blenda

**Affiliations:** 1 Department of Biomedical Sciences, University of South Carolina School of Medicine Greenville, 607 Grove Rd., Greenville, SC 29605, USA; asansone@email.sc.edu (A.M.S.); bhisrich@email.sc.edu (B.V.H.); youngrb@email.sc.edu (R.B.Y.); wabel@email.sc.edu (W.F.A.); bergmanb@email.sc.edu (B.B.B.); averytf@email.sc.edu (A.T.F.); 2 Department of Obstetrics and Gynecology, Prisma Health Upstate, 701 Grove Rd., Greenville, SC 29605, USA; zbowens@carolinas.vcom.edu (Z.B.); lisa.green2@prismahealth.org (L.J.G.); 3 Pathology Associates of Greenville, 701 Grove Rd., Greenville, SC 29605, USA; david.schammel@prismahealth.org; 4 Department of Obstetrics and Gynecology, Wake Forest University Baptist Hospital, 1 Medical Center Blvd, Winston-Salem, NC 27157, USA; blessey@wakehealth.edu

**Keywords:** endometriosis, SIRT1, BCL6, ELISA, non-invasive, infertility, laparoscopic surgery, biomarker

## Abstract

(1) Background: Endometriosis is characterized by the presence of endometrial glands and stroma outside of the uterus and is often associated with severe pelvic pain and infertility. Our study explored the utilization of B-Cell Lymphoma 6 (BCL6) and Sirtuin 1 (SIRT1) as potential biomarkers in serum, plasma, urine, and cervical mucus for a non-invasive diagnostic test for endometriosis. BCL6 was chosen based on its previously reported elevated expression in endometrial biopsies, and SIRT1 is co-expressed and upregulated in the endometrium of women with endometriosis. (2) Methods: BCL6 and SIRT1 levels were measured using enzyme-linked immunoassay (ELISA) in samples from 20 women with endometriosis (ten with stages I/II and ten with stages III/IV) and ten women without endometriosis. (3) Results: Levels of SIRT1 in sera showed a statistically significant elevation in advanced stages III/IV compared to controls and stages I/II. No significant differences were found in other bodily fluids for SIRT1 or any bodily fluids tested for BCL6. (4) Conclusions: These results suggest some potential of SIRT1 expression within serum as a predictor of advanced asymptomatic stages of endometriosis. Using immunohistochemistry (IHC) staining and H-SCORE values for the elevated BCL6 (and potentially SIRT1) levels in endometrial biopsy samples seems to have higher diagnostic potential based on the previously published studies.

## 1. Introduction

Endometriosis is a chronic inflammatory disease characterized by the presence of endometrial glands and stroma outside of the uterine cavity [1]. The presence of these ectopic cells has often been associated with severe pelvic pain and infertility [2]. It is estimated that 10–15% of reproductive age women are affected by endometriosis, and that 70% of women presenting with chronic pelvic pain are subsequently diagnosed with endometriosis [3,4]. The typical method for endometriosis diagnosis is surgical, requiring visual evidence that can be biopsied for pathologic confirmation [5]. Although the disease is well-known and has established diagnostic criteria, the average time between the onset of symptoms and diagnosis of endometriosis is 6.7 years [6]. The burden of endometriosis can be mentally, emotionally, and physically taxing. Women who struggle with endometriosis face a decreased quality of life due to chronic pain, infertility, numerous hospitalizations, missed workdays, and often undergo a barrage of surgeries and radiographic imaging studies [7]. The annual healthcare cost combined with the cost of associated productivity loss was extrapolated to be $22 billion in the United States in 2002 [8]. A more recent retrospective study showed that patients diagnosed with endometriosis incurred significantly higher direct and indirect healthcare costs compared to matched healthy controls [9].

Although usually diagnosed in the early 20s, endometriosis symptoms can present in early adolescence and carry a high economic and social burden. First-line treatment of sis symptoms is medical management [10], with definitive diagnosis in the case of continued symptoms refractory to medical management [11]. During this medical management phase, patients describe switching doctors or trying multiple therapies without a true diagnosis of endometriosis.

**Figure 1:**
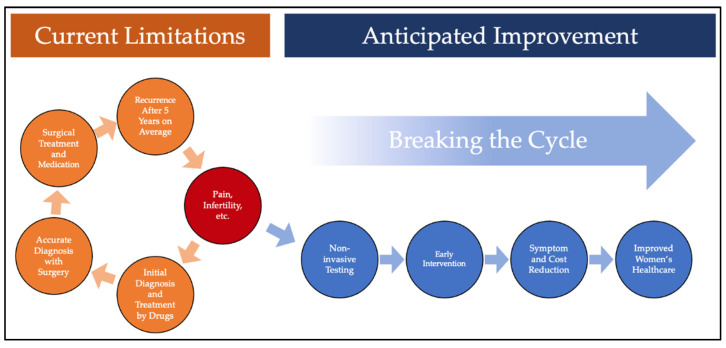
Current limitations of endometriosis diagnosis and anticipated improvement with the development and use of reliable non-invasive testing.

This project aims to determine if differential expression of B-Cell Lymphoma 6 (BCL6) and Sirtuin 1 (SIRT1) proteins can be used as a marker to aide in the diagnosis of endometriosis through less invasive means. This type of biomarker(s) would offer a less invasive diagnostic tool for clinicians, which could assist with identifying patients with endometriosis sooner (Figure 1).

Although the pathogenesis of endometriosis is unknown, there are several theories suggesting explanations as to the disease progression and inheritance [12,13]. A highly plausible mode of endometriosis spread is through retrograde menstruation [14,15]. Another potential cause is the localized response to estrogen receptor stimulation in the pelvis area [16]. Progesterone resistance is now thought to be part of the pathogenesis of endometriosis [17,18,19,20].

We recently described the association of BCL6 and SIRT1 as potential mediators of progesterone resistance in women with endometriosis [21]. Additionally, since estrogen is a mitogen, the proximity and localized secretion of estrogen from the ovaries increases the proliferation of these cells and the progesterone resistance of these cells blunt the anti-mitogenic and apoptotic effect of progesterone [20,22,23]. Ectopic endometriosis cells are known to display a genetic profile of increased proliferation markers and decreased apoptotic markers [18,24]. These characteristics explain the aggressive behavior of those cells in women with endometriosis compared to women who do not have the disease.

The BCL6 protein is encoded by the *BCL6* oncogene. BCL6 is a zinc finger transcription factor and contains an N-terminal Pox virus and zinc finger (POZ) domain [25,26]. BCL6 functions as a sequence-specific transcription repressor and has been shown to regulate the transcription of signal transducer and activators of transcription (STAT)-dependent IL-4 responses of B-cells [27]. The *BCL6* gene is often translocated and mutated in diffuse large-cell lymphoma (DLCL) and may be involved in the pathophysiology of DLCL as well as other non-Hodgkin’s Lymphomas [28,29,30]. Using immunohistochemical staining, BCL6 is shown to be present intracellularly within both the nuclei of endometrial epithelial cells and B-cells in lymph node germinal centers [31,32]. Furthermore, BCL6 and SIRT1 are over-expressed in the eutopic endometria of women with endometriosis. They likely contribute to the progesterone resistance of the endometrium and thus estrogen dominance that supports the proliferation of endometrial cells [21].

SIRT1 protein, also known as nicotinamide adenine dinucleotide (NAD)-Dependent Protein Deacetylase Sirtuin-1, is a member of the class 1 sirtuin family of proteins. This family of proteins is characterized by a sirtuin core domain and is divided into four classes [33]. The *SIRT1* gene, located on chromosome 10 in humans, is a homolog of yeast *Sir2* which is known to regulate epigenetic modifications and rDNA recombination [34]. Previous studies suggest a similar gene silencing and chromosomal stabilization mechanism of human sirtuin proteins via mono-ADP ribosylation [35,36]. In mammals, SIRT1 has been shown to deacetylate, and thus inactivate, p53, an important tumor suppressor that regulates the cell cycle [37,38,39]. SIRT1 is involved in the regulation of cell divisions, aging, and metabolism [40,41] and can function as both a tumor suppressor and an oncoprotein [42]. SIRT1 has been found to be overexpressed in the endometrium of women with endometriosis and co-expressed with BCL6 [21,43]. For this reason, SIRT1 protein was investigated as a potential diagnostic biomarker for endometriosis.

**Figure 2:**
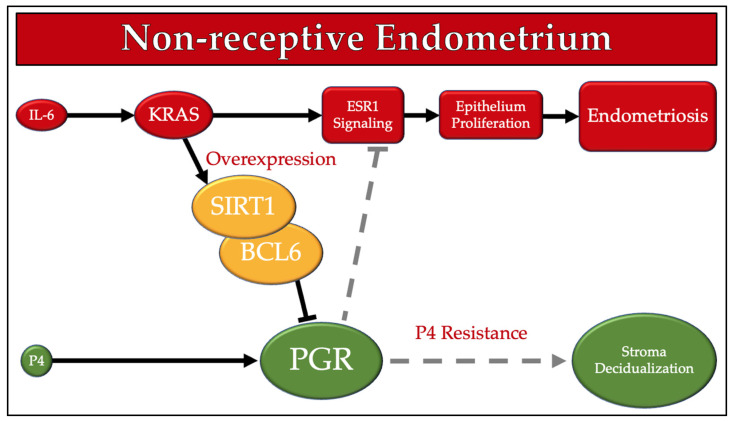
Role of SIRT1 and BCL6 in Endometriosis. Increases in inflammatory responses, such the inflammatory cytokine IL-6, activates the oncogene *KRAS* to induce both SIRT1 and BCL6 production. SIRT1 and BCL6 are both thought to interfere with progesterone signaling, leading to progesterone resistance and estrogen dominance leading to endometriosis proliferation as described in Yoo (2017) and Marquardt (2019). BCL6, B-Cell Lymphoma 6 protein; ESR1, Estrogen Receptor 1; IL-6, Interleukin 6; KRAS, Kirsten Rat Sarcoma Viral Oncogene Homolog; P4, Progesterone; PGR, Progesterone Receptor; SIRT1, Sirtuin 1.

Figure 2 shows SIRT1 and BCL6 and a simplified version of their involvement in dysregulated eutopic endometrial tissue based on previous descriptions of their interactions [21,23].

Since both SIRT1 and BCL6 are differentially expressed in the eutopic endometrium of women with endometriosis [21], these two proteins were chosen for our study using ELISA assay of bodily fluids including serum, plasma, urine, saliva, and cervical swab. The goal of our study was to determine if levels of SIRT1 and/or BCL6 were differentially expressed in any of these bodily fluids and could potentially be used as part of a less invasive diagnostic test for the presence of endometriosis.

## 2. Materials and Methods

### 2.1. Sample Collection and Preparation

Patients included in the study were within reproductive age, had regular menstrual cycles, and could not have a previous diagnosis of polycystic ovarian syndrome (PCOS) or take hormonal birth control (Table 1).

Subjects that satisfied the above inclusion and exclusion criteria were consented prior to undergoing benign gynecological surgery (Table 2) at the Prisma Health Upstate, Department of Obstetrics and Gynecology, Greenville, SC, USA. All surgeries were either laparoscopic or laparoscopic with hysteroscopy.

During the surgery, a study team member collected normal eutopic endometrial tissue, tissue that appeared to be concerning for endometriosis, whole blood, cervical mucus, and urine samples with the assistance of the surgical team. Control patients were identified as “endometriosis negative,” and endometriosis patients were further separated based on American Society of Reproductive Medicine (ASRM) staging, with stages I and II representing minimal/mild disease and stages III and IV representing moderate/severe disease. In many attempts to obtain a negative control sample the patient was found to have evidence of endometriosis during the surgery which then automatically re-categorized that patient as “endometriosis positive.” Samples were staged by surgical determination as I/II or III/IV based on the burden of disease presence. Thus, stages I and II and stages III and IV were combined in our data analysis.

### 2.2. Sample Processing and Storage

One plasma and two serum Vacutainers (BD, Franklin Lakes, NJ, USA) were collected per patient. All three tubes were placed on ice immediately. Upon arrival to the laboratory, the plasma Vacutainer was spun immediately after collection at 3000 rpm for 10 min, and 0.5 mL of the supernatant aliquot were placed into micro-centrifuge tubes. The tubes were labeled, frozen in liquid nitrogen, and stored in a −80 °C freezer.

The serum Vacutainers remained on ice for 30 min following collection to allow for clotting prior to spinning the Vacutainers at 3000 rpm for 10 min. After spinning, the clear coagulant was carefully pushed to the bottom of the Vacutainer with a sterile pipet tip. It was then spun again at 3000 rpm for 3 min, and 0.5 mL of the supernatant was aliquoted into micro-centrifuge tubes.

The tubes were labeled with the study number, the sample type (plasma, serum, urine, or cervical swab), the date of collection, and whether the sample was collected pre- or post-operation. Next, the tubes were frozen in liquid nitrogen and stored in the −80 °C freezer.

For urine sample processing, 10 mL of urine were placed in a sterile tube for centrifugation and spun at 3000 rpm for 10 min. After spinning, 1 mL of urine was placed in each microcentrifuge tube. Only 5 microcentrifuge tubes were filled with urine per patient participant. The tubes were labeled, frozen in liquid nitrogen, and stored in the −80 °C freezer.

For cervical swab sample processing, 1X RIPA Lysis Buffer solution was prepared from 10X stock (MilliporeSigma, Burlington, MA, USA) prior to sample collection in the operating room and placed in a microcentrifuge tube. After sample collection, the cervical swab brushes were placed into the prepared 1X RIPA Lysis Buffer with swirling of the brush to introduce cervical mucus into the solution and immediately placed on ice in the operating room. Once in the laboratory, the 1X RIPA buffer sample was centrifuged at maximum speed for 15 min. Then, 375 µL of the 1X RIPA buffer sample were aliquoted into 4 microcentrifuge tubes. The tubes were labeled, frozen in liquid nitrogen, and stored in the −80 °C freezer.

### 2.3. ELISA Procedure

Aviva Systems Biology (San Diego, CA, USA) SIRT1O ELISA kit (Human-OKEH01724; limit of detection 32 pg/mL) and protocol were used for all SIRT1 ELISA procedures for serum, plasma, urine, saliva, and cervical mucus samples collected. MyBioSource (San Diego, CA, USA) Human B-Cell Lymphoma 6 (BCL6) ELISA Kit (sensitivity 1.0 pg/mL) and protocol were used for all BCL6 ELISA procedures for serum, plasma, urine, saliva, and cervical mucus samples. One-to-one dilutions of patients’ samples were tested for SIRT1 and BCL6 levels to establish a fit within their respective standardized curves. All ELISAs were performed in duplicate at minimum for each sample. The ELISA microplate was analyzed at 450 nm to obtain optical density (OD) values. Both protocols included guidelines for the calculations of the results, which were followed for SIRT1 and BCL6 ELISA procedures. A standard curve was derived from the OD readings of standards with known concentrations, and the unknown sample concentrations were determined from the standard curve.

### 2.4. Data Analysis

JMP^®^ 15 (SAS, Cary, NC, USA) was used to perform unpaired, independent *t*-tests to determine statistical significance of the differences between the average concentration of SIRT1 and BCL6 in negative controls and patients with endometriosis. Descriptive statistics and ROUT analysis with a Q of 10% were used to confirm that there were no outliers in stages I/II or III/IV that would skew the results. Graphs were produced to reflect the individual data points and depict the statistical significance or lack thereof between the groups. A power analysis was performed, and the sample size was determined to sufficient for the study.

## 3. Results

Study population age and race are shown in Table 3. There was no association of age with the severity of endometriosis. The average patient age was 29.7 years for negative controls, 34.5 years for endometriosis stages I/II, and 31.6 years for endometriosis stages III/IV.

### 3.1. BCL6 ELISA

**Figure 3:**
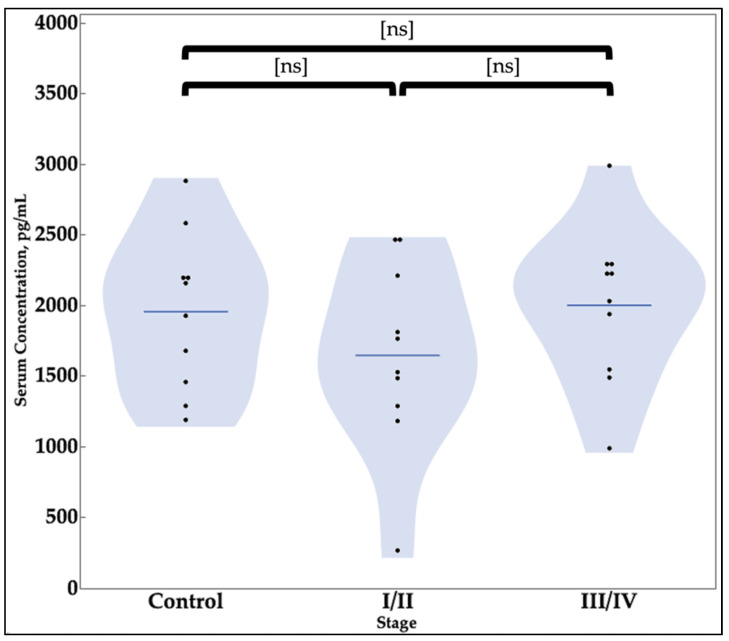
Comparison of Serum Concentrations of BCL6. No statistically significant difference is observed in the serum levels of BCL6 in healthy individuals compared to patients with I/II or III/IV Endometriosis. The “Control” group is patients with no endometriosis. The groups “Stage I/II” and “Stage III/IV” contained samples with stages I or II and stages III or IV endometriosis, respectively (ns = not significant).

The average BCL6 concentration in serum for patients without endometriosis was 1953.8 pg/mL with a sample size of ten (Table 4). The average serum concentration of BCL6 in serum for patients with stages I or II of endometriosis was 1645.7 pg/mL with a sample size of ten. In patients with stage III or IV endometriosis, the average concentration was 2000.5 pg/mL, with a sample size of ten. No statistically significant differences in BCL6 levels were found between patients with endometriosis and patients without endometriosis. Further, no differences in BCL6 levels were found when stages I and II were compared to stages III and IV (Table 4 and Figure 3).

### 3.2. SIRT1 ELISA

**Figure 4:**
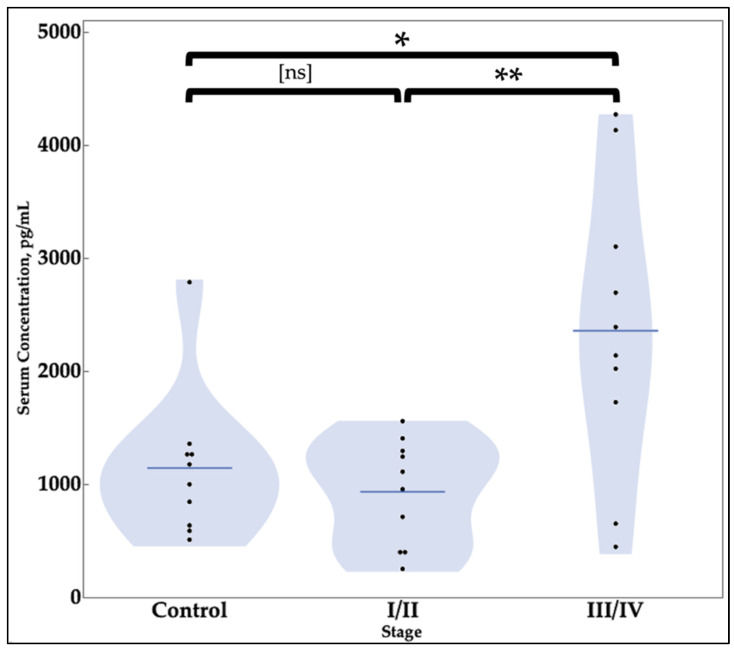
Comparison of Serum Concentrations of SIRT1. A statistically significant difference is observed in the serum levels of SIRT1 in stage III/IV endometriosis patients compared to stage I/II endometriosis patients and healthy individuals. The groups “Stage I/II” and “Stage III/IV” contained samples with stages I or II and stages III or IV endometriosis, respectively. There was a statistically significant difference between the group “Stage I/II” and “Stage III/IV” with a *p*-value of 0.0038. There was also a statistically significant difference between the “Control” and “Stage III/IV” with a *p*-value of 0.0152. (ns = not significant, * *p* ≤ 0.05, ** *p* ≤ 0.01).

The average SIRT1 concentration in sera for patients without endometriosis was 1141.6 pg/mL with a sample size of ten (Table 4). The average serum concentration of SIRT1 in patients with stages I and II (combined) of endometriosis was 932.3 pg/mL with a sample size of ten (Table 4). The average serum concentration of SIRT1 in patients with stages III and IV of endometriosis was 2357.5 pg/mL with a sample size of ten. When the average SIRT1 serum concentration in patients with stages I and II was compared to the average SIRT1 concentration from patients with stages III and IV endometriosis, a *t*-test resulted in a *p*-value of 0.0038, indicating a significant difference. Comparing SIRT1 values between patients with stages I and II of endometriosis with the control patient group resulted in a *p*-value of 0.4213. Finally, comparison of serum SIRT1 levels from patients with no endometriosis with serum from patients with stage III or stage IV endometriosis resulted in a *p*-value of 0.0152 (Table 4 and Figure 4).

## 4. Discussion

In searching for a less invasive diagnostic tool for endometriosis, our investigation built upon previous work that demonstrated increased levels of BCL6 in eutopic endometria of patients with endometriosis compared to endometria without endometriosis [32]. The aforementioned study showed that BCL6 expression was significantly increased during the secretory phase of menstruation for women with endometriosis as compared to negative control women. Analysis of these expression differences resulted in the development of an H-SCORE immunohistochemistry cutoff of >1.4 to define the difference in BCL6 expression between women with endometriosis and those without. Previous work establishing BCL6 expression within the endometrium was based on the idea that endometriosis is associated with inflammation which includes upregulation of inflammatory cytokines. In addition, studies suggest that IL-6 is upregulated in endometriosis patients, which leads to downstream signaling of pSTAT3 and BCL6 [32,44,45,46,47]. Thus, a correlation between endometriosis, inflammatory cascades, and subsequent elevation in BCL6 levels was suggested.

Our exploration of BCL6 and SIRT1 levels in bodily fluids take the above-described investigation one step further. The current diagnostic guidelines for endometriosis require invasive laparoscopic surgery. Using eutopic endometrial biopsies is substantially less invasive than laparoscopic surgery, but it is still invasive and painful. However, if the endometrial overexpression of BCL6 and SIRT1 in patients with endometriosis could be extended to levels of BCL6 and SIRT1 in their serum or plasma, the diagnosis of the disease could be made significantly less invasive and more accessible to patients.

However, when looking at the results obtained for the BCL6 serum ELISA values, there were no statistically significant differences between healthy controls and any stages of endometriosis, even though BCL6 has been shown previously to be elevated in the endometrium of women with endometriosis [32].

As previously discussed, the pathogenesis of SIRT1 over-expression is similar to the BCL6 inflammatory cascade, which led to our investigation targeting both BCL6 and SIRT1 proteins. While both BCL6 and SIRT1 were analyzed in all the bodily fluids collected, it was not possible to receive meaningful results from all non-invasive bodily fluids. Cervical swab results frequently yielded minimal and unreliable results, with most concentrations of BCL6 and SIRT1 not detectable. Urine similarly yielded minimal and unreliable results that also were not detectable. While the urine and cervical swabs are less invasive than a blood draw for serum or plasma, the utility of sampling must be matched with the reliability of the results. In terms of using plasma in this investigation, statistically significant results were found with serum sampling rather than plasma. Since these samples required the same level of invasiveness for collection, serum remained the more promising future diagnostic bodily fluid given the potentially higher diagnostic value of the results compared to plasma.

The elevation of SIRT1 serum levels in stages III/IV follows the prediction by earlier studies of endometriosis which anticipated a rise in markers of inflammatory cascade such as BCL6 and SIRT1 [21]. While there was an initial decrease in SIRT1 levels noted for women with endometriosis stages I/II, the expected upregulation of SIRT1 was found to have occurred with increased disease severity in stages III/IV (Figure 3). This points towards the possible utility of serum SIRT1 levels as a predictor of advanced endometriosis that may not be associated with severe symptoms or pain, rather than an initial screening for the disease itself. The future utility of SIRT1 as a marker for endometriosis needs to be tested further with increased sample sizes for both negative controls and women with endometriosis of various stages.

We also noticed that the variance of SIRT1 in stage III/ IV was relatively high. Perhaps, if SIRT1 really is elevated with the progression of endometriosis stage, by looking at increased sample sizes of stages III and IV separately, we might find an even higher mean and better significance when comparing them separately to controls than when comparing controls and stages III and IV combined.

In terms of clinical limitations within this study, there were several criteria that needed to be met by patients to be included in this investigation. In order to have an equal comparison between disease state and non-disease states, ideal negative controls were patients that had no significant medical conditions, were not on oral contraceptive pills so that they had natural menstrual cycles, were of reproductive age, and were undergoing gynecological (laparoscopic) surgery in which samples for our investigation could be collected. This was a challenging set of criteria to meet given that oral contraceptive pills are the most widely used form of birth control in the United States and our study focused on reproductive age women, and a number of those undergo gynecological surgery in order to investigate pelvic pain of some kind. Multiple clinical limitations determined smaller sample sizes used in the study and, as a result, larger sample size studies are warranted.

## 5. Conclusions

Based on the results of this exploratory study, the feasibility of using BCL6 and SIRT1 as protein markers in bodily fluids (such as serum, cervical swab, and urine) for a non-invasive diagnosis of endometriosis is relatively low. The potential of SIRT1 marker for advanced asymptomatic endometriosis needs to be investigated further with larger patient sample size. Using immunohistochemistry staining and H-SCORE values for the elevated BCL6 (and potentially SIRT1) levels in the endometrial biopsy samples seems to have higher diagnostic potential based on the previously published studies. Obtaining biopsy samples is more intrusive than obtaining the above-mentioned bodily fluids; however, biopsy samples collected during regular gynecological visits are still a significant step forward on the road to development the non-invasive diagnostics for endometriosis.

## Figures and Tables

**Table 1:** Criteria for Patient Selection.

Categories	Inclusion Criteria	Exclusion Criteria
Age, years	18–42	>42
Menopausal Status	Premenopausal	Postmenopausal
Menstruation Status	Cycle frequency 21–35 days	Cycle < 21 days or >35 days
Past Medical History *	NA	PCOS, psychiatric history, diabetes, known communicable infections
Medications	NA	Hormonal therapy in the past 3 months
Pregnancy Status	None	Pregnant
Surgery Type	Gynecological **	Oncologic

* Prior diagnosis of endometriosis was an exclusion criterion for the selection of negative controls. ** See Table 2 for surgery details.

**Table 2:** Gynecological Surgeries Used for Sample Collection.

Surgery, Count	Control Group	Stage I/II	Stage III/IV
Adhesion Lysis	0	0	2
Adnexal Removal	1	0	0
Chromotubation	0	1	1
Dilation and Curettage	0	0	1
Endometriosis Resection	2	6	3
Hysterectomy	1	2	1
Myomectomy	0	3	0
Ovarian Cystectomy	1	0	2
Oviduct Fulguration	2	0	0
Salpingectomy	2	0	0

**Table 3:** Demographics of Study Population.

Characteristic	Control	I/II	III/IV
Age, mean (SD)	29.7 (4.9)	34.5 (5.8)	31.6 (3.5)
Race, %			
African American	40	20	0
Asian	0	0	10
White	50	60	90
White/Hispanic	0	10	0
Hispanic	10	0	0
Unknown	0	10	0

SD = standard deviation.

**Table 4:** Average Concentrations of BCL6 and SIRT1 Proteins in Patient Serum Samples.

Value Category	SIRT1	BCL6
Concentration—pg/mL, mean (SD) *		
Negative Controls	1141.7 (655.4)	1953.8 (554.0)
Stage I/II Endometriosis	932.3 (466.3)	1645.7 (665.9)
Stages III/IV Endometriosis	2357.5 (1274.5)	2000.5 (552.4)
*t*-test Results		
Negative Controls vs. Stages I/II	0.4213	0.2754
Negative Controls vs. Stages III/IV	0.0152 **	0.8525
Stages I/II vs. Stages III/IV	0.0038 **	0.2111

* SD = standard deviation. ** *t*-test results yielded a *p*-value of less than 0.05 indicating statistical significance.

## Data Availability

All data are available in the manuscript.
